# Automatic Censoring CFAR Detector Based on Ordered Data Difference for Low-Flying Helicopter Safety

**DOI:** 10.3390/s16071055

**Published:** 2016-07-08

**Authors:** Wen Jiang, Yulin Huang, Jianyu Yang

**Affiliations:** School of Electronic Engineering, University of Electronic Science and Technology of China, Chengdu 611731, China; yulinhuang@uestc.edu.cn (Y.H.); jyyang@uestc.edu.cn (J.Y.)

**Keywords:** low-flying helicopter safety, nonhomogeneous clutter environment, constant false alarm rate (CFAR), first order difference (FOD)

## Abstract

Being equipped with a millimeter-wave radar allows a low-flying helicopter to sense the surroundings in real time, which significantly increases its safety. However, nonhomogeneous clutter environments, such as a multiple target situation and a clutter edge environment, can dramatically affect the radar signal detection performance. In order to improve the radar signal detection performance in nonhomogeneous clutter environments, this paper proposes a new automatic censored cell averaging CFAR detector. The proposed CFAR detector does not require any prior information about the background environment and uses the hypothesis test of the first-order difference (FOD) result of ordered data to reject the unwanted samples in the reference window. After censoring the unwanted ranked cells, the remaining samples are combined to form an estimate of the background power level, thus getting better radar signal detection performance. The simulation results show that the FOD-CFAR detector provides low loss CFAR performance in a homogeneous environment and also performs robustly in nonhomogeneous environments. Furthermore, the measured results of a low-flying helicopter validate the basic performance of the proposed method.

## 1. Introduction

Helicopters, used in law enforcement, fire fighting, medical evacuation, civil emergencies, search and rescue and new/traffic reporting, are often required to fly at low altitudes of limited visibility [1,2]. Obstacles in the flight path or close to it often pose a significant threat to low-flying helicopters [3]. Such obstacles may be power lines, aerial cableways, pylons and towers [3,4]. The absence of adequate sensing and warning equipment for low-flying helicopters has proven to be of disastrous consequence [5,6,7,8]. Many researchers developed optical sensors to improve the safety of low-flying helicopters [1,3,4]. However, optical sensors are highly sensitive to atmospheric conditions, such as clouds, fog, smoke, dust and precipitation [9,10]. Radar provides robustness to atmospheric conditions, and it is widely used in a low-flying helicopters to sense the surrounding environment [2,10,11,12,13,14,15,16]. Automatic detection of obstacles would help conserve the pilot’s attention for the mission tasks, thus contributing to mission success and, most importantly, saving lives [4]. This article focuses on radar signal detection algorithm of low-flying helicopters.

Since the clutter and noise power are usually unknown, fixed threshold detection schemes cannot be applied in the radar of low-flying helicopters. This calls for a class of constant false alarm rate (CFAR) detection schemes whose threshold is calculated adaptively, and false alarm rate performance is almost independent of the background clutter and noise power. The cell-averaging CFAR (CA-CFAR) [17] is often used for adaptive threshold generation in radar systems. If the outputs of the references cells are statistically independent and identically distributed exponential random variables, the detection performance of the CA-CFAR detector is optimum as the number of reference cells becomes very large [18,19].

In a real low-flying helicopter application scenario, however, two cases of nonhomogeneous environments are often encountered. The first situation of nonhomogeneous environments is often modeled as multiple targets, where one or more large discrete and spiky clutters, such as power lines, towers and steep mountains terrain, are presented in the reference window [20]. The second situation of nonhomogeneous environments is modeled as clutter edges, which occurs as a consequence of weather change, jamming, mountainous areas with rapid elevation/reflectivity variation and rapid land cover variations [20,21]. Therefore, when the clutter background environment is nonhomogeneous, such as multiple targets and clutter edges, the performance of the CA-CFAR detector is seriously degraded, leading to excessive false alarms or target masking [22,23,24,25,26,27,28,29,30]. If excessive false alarms occur, the low-flying aircraft will be encumbered by unnecessary obstacle avoidance. Meanwhile, if target masking occurs in radar signal detection, it can result in a catastrophic collision accident.

Several variations of the CA-CFAR detectors called mean-level detectors have been proposed in order to mitigate the impact of nonhomogeneous clutter environments. The smallest-of CFAR (SO-CFAR) [31] offers better performance, reducing target masking in a multiple target environment, but sacrifices the CFAR behavior in the clutter edge environment. The greatest-of CFAR (GO-CFAR) [32] offers better performance than CA-CFAR in the case of a clutter edge, but has increased loss in a multiple target environment. In [21], Smith and Varshney presented the concept of the variability index (VI) detection. The VI-CFAR exhibits a low-loss CFAR in a homogeneous background and performs robustly in nonhomogeneous environments [33]. However, its detection performance degrades if the interfering targets are not confined to a single half of the reference window [21].

Besides, a class of order statistic-based thresholding schemes has been proposed to overcome the performance limitations of the CA-CFAR and its variations. In [34], Rohling proposed the order statistic CFAR (OS-CFAR) schemes, which rely on the power ranking of the reference cells. Nevertheless, the performance of OS-CFAR suffers from excessive false alarms during clutter transitions [35]. It is also shown in [35] that OS-CFAR can resolve the primary target from up to *k* interfering targets. Rickard and Dillard [36] introduced the censored mean level detector (CMLD), where some largest range cells are censored from the reference window. Afterward, a generalization of the OS-CFARAand the CMLD, known as the trimmed mean (TM-CFAR) detector [35], has been proposed. In CMLD and TM-CFAR, the censoring point is preset. This implies that some a priori knowledge about the background environment is needed in order to censor the unwanted samples [37,38]. To evade this problem, Himonas and Barkat [37] introduced the automatic CMLD (ACMLD) and generalized two-level censored mean level detector (GTL-CMLD), which do not require any prior information about the environment. The ACMLD and the GTL-CMLD are based on the same cell-by-cell procedure for rejecting the unwanted cells and suffer from substantial computational requirements [38]. Then Farrouki and Barkat [38] proposed an automatic censored cell averaging (ACCA) CFAR detector based on ordered data variability (ODV) for nonhomogeneous background environments. The ACCA-ODV detector exhibits small detection loss in homogeneous environments and performs robustly in the presence of interference in the reference window. In [39], the authors introduced a automatic dual censoring cell averaging (ADCCA)-CFAR detector that uses the membership function of the OS processor and two threshold values to determine and censor the unwanted samples in the reference window. In [40], Boudemagh and Hammoudi presented an automatic censoring (AC)-CFAR detector for heterogeneous Gaussian clutter. The proposed detector dynamically switches to the optimal conventional detector CA-, CMLD- or TM-CFAR. The AC-CFAR exhibits a low-loss CFAR in a homogeneous background and performs with considerable robustness in the presence of interfering targets and/or clutter edge situations.

In this paper, we develop a new CFAR detector operating in a nonhomogeneous clutter environment. The proposed CFAR detector does not require any prior information about the background environment and uses the hypothesis test of the first-order difference (FOD) result of ordered data to reject the unwanted samples in the reference window. After censoring the unwanted ranked cells, the remaining samples are combined to form an estimate of the background power level, thus getting better radar signal detection performance of low-flying aircraft. As FOD is the main feature of this algorithm that distinguishes it from other CFAR detectors, we call it FOD-CFAR.

The paper is organized as follows. In Section 2, the target detection model of a low-flying helicopter is introduced. In Section 3, the FOD-CFAR concept is detailed. Section 4 describes the procedure by which the FOD-CFAR parameters are determined. The FOD-CFAR performance analysis results for various environments are summarized in Section 5. Detection analysis for Swerling II targets and single pulse processing is presented in this paper. Meanwhile, measured data validate the basic performance of FOD-CFAR. Section 6 summarizes and concludes the paper.

## 2. Target Detection Model of a Low-Flying Helicopter

The main objective of a low-flying helicopter is to maintain a relatively fixed altitude above the terrain without hitting anything. However, there are a wide variety of ground-based obstacles that present a hazard to low-flying helicopters. The need to detect obstacles in the flight path and to alert the pilot in time to take corrective measures is critical to the safety of the helicopter. Many systems first generate a trajectory to the mission destination based on a digital terrain elevation database and then control the helicopter to follow the trajectory within maneuvering capabilities. However, the terrain elevation database is not updated very quickly when new features arise, and it contains some systematic errors; other real-time measurement methods are also required [2,5]. Because optical sensors are highly sensitive to atmospheric conditions, such as clouds, fog, smoke, dust and precipitation, radar is widely used in a low-flying aircraft to sense the surrounding environment.

**Figure 1:**
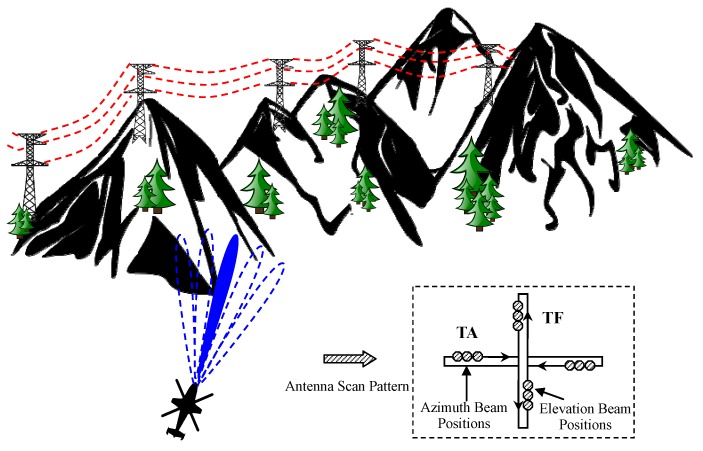
Target detection model of a low-flying helicopter.

The working principle and target detection model of a low-flying helicopter is shown in Figure 1. There are two radar antenna scan patterns used in a low-flying helicopter. In TF, the antenna scans several vertical bars oriented along the helicopter velocity vector and generates an altitude-range profile that is displayed to the pilot. In TA, the antenna scan is in a horizontal plane. Several altitude plane cuts are estimated and presented to the pilot [6]. The object of the TF scan helps aircraft to fly over obstacles, whereas TA scan flies around obstacles. In both the TF and TA scan, one main task of the radar is to detect obstacles, including man-made structures, towers, power lines, etc. [5].

Since the clutter and noise power are usually unknown, adaptive threshold techniques must be adopted in radar signal detection of a low-flying helicopter. Meanwhile, nonhomogeneous environments, such as multiple targets and clutter edges, are easily encountered in real application scenarios. These limitations seriously affect the detection performance of traditional detectors. To obtain a better detection performance in these cases, we develop the FOD-CFAR detector.

## 3. FOD-CFAR Processor

### 3.1. Idea Behind FOD-CFAR

For each test cell, the CFAR processor makes a target present/absent decision based on a comparison of the value of the test cell to an adaptive threshold. The adaptive threshold is defined as the product of a background multiplier constant and a background noise/clutter power estimate. The uniqueness of FOD-CFAR lies in how the background power estimation is performed.

It is generally known that the FOD result describes the variation of adjacent data. After ordered samples from a homogeneous environment are computed by the FOD, we assumed that the FOD results will not exceed an explicit threshold with a certain error probability. If the results exceed the threshold, it means that interference samples from a nonhomogeneous environment exist in the ordered samples. Thus, after censoring the interference samples in the ordered data, we can obtain a better performance of background power estimation.

**Figure 2:**
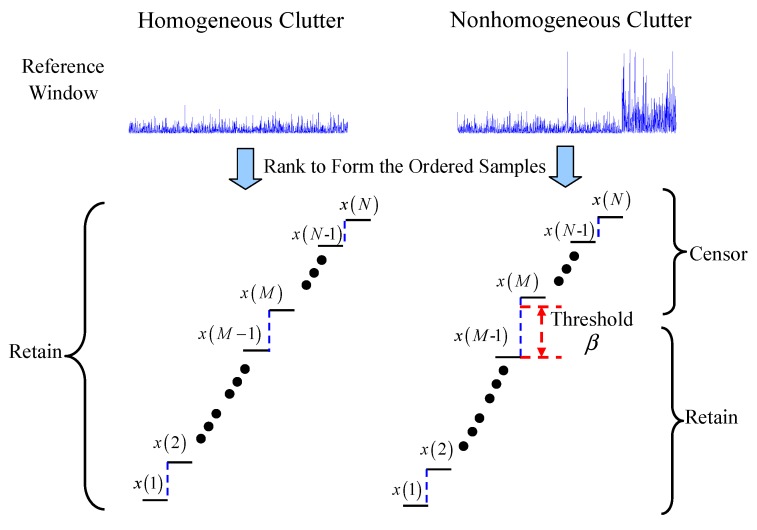
The idea behind first-order difference (FOD)-CFAR.

Figure 2 shows the idea of FOD-CFAR. The *N* reference cells are first ranked to form the ordered samples. In a homogeneous environment, it is shown that none of the FOD results of ordered data have exceeded the explicit threshold *β*. Then, all of the ordered data are retained. In a nonhomogeneous environment, the difference between x(M-1) and x(M) has exceeded the threshold *β*. Thus, the ordered cells being greater than x(M-1) are censored from reference window, and the remaining M-1 samples are reserved.

Based on the above analysis and conclusion, the proposed FOD-CFAR detector first uses the hypothesis test of FOD results of ordered data to reject the interference samples in the reference window and then utilizes the remaining reference cells to calculate the background power.

### 3.2. Description of the FOD-CFAR Algorithm

**Figure 3:**
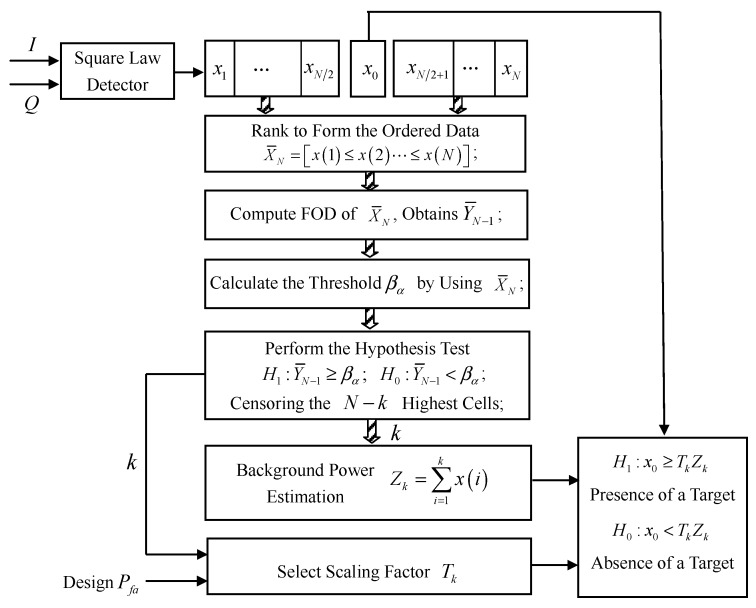
FOD-CFAR block diagram.

The FOD-CFAR processor block diagram is provided in Figure 3. In-phase and quadrature (*I* and *Q*) signals correspond to samples of radar time/range returns from a matched filter receiver. The *I* and *Q* signals are first square-law envelope detected. Then, the N+1 square-law detected samples are sent serially into a tapped delay line. The N+1 samples correspond to a test cell $x_{0}$ centered in *N* samples of the reference cell. For a homogeneous noise/clutter environment, the *I* and *Q* input signals are assumed to be independent and identically distributed (IID) Gaussian random processes with zero mean. Consequently, the envelope amplitude at the output of a square-law detector is also an IID process with an exponentially distributed random variable.

The FOD-CFAR algorithm can be described as follows.

1. The reference cells are first ranked to form the ordered samples:
  (1) $${\bar{X}}_{N}=\left\{x\left(i\right),i=1,2,\ldots ,N\;and\;x\left(1\right)\leq x\left(2\right)\leq \cdots \leq x\left(N\right)\right\}$$
2. After computing the first-order forward difference of ${\bar{X}}_{N}$, we obtained:
  (2) $${\bar{Y}}_{N-1}=\left\{y\left(i\right)=x\left(i+1\right)-x\left(i\right),i=1,2,\ldots ,N-1\right\}$$
3. We use *p* lowest cells of ordered samples ${\bar{X}}_{N}$ to calculate the threshold $\beta_{\alpha }$. The implementation method of $\beta_{\alpha }$ is described in the next section.
4. Then, ${\bar{Y}}_{N-1}$ is compared to the corresponding threshold $\beta_{\alpha }$, and a binary decision is made in favor of $H_{1}$ or $H_{0}$ according to the hypothesis test:
  (3) $$H_{1}:{\bar{Y}}_{N-1}\geq \beta_{\alpha };H_{0}:{\bar{Y}}_{N-1}<\beta_{\alpha }$$
  Hypothesis $H_{1}$ corresponds to the case where the reference cell is declared nonhomogeneous, while $H_{0}$ denotes the hypothesis where the reference cell is declared homogeneous. While the hypothesis $H_{1}$ is true, we find ${\bar{Y}}_{N-1}\left(k\right)$, which is greater than $\beta_{\alpha }$. Then, we decide that the ordered subset [x(1)≤x(2)≤⋯≤x(k)] corresponds to homogeneous returns. After that, the N-k highest cells will be censored. In the censoring process, *k* corresponds to the estimated number of samples to be reserved in the reference window, and N-k denotes the estimated number of samples to be censored from the upper end of ordered samples ${\bar{X}}_{N}$.
5. After censoring the unwanted ranked cells, the remaining *k* samples are combined to form an estimate $Z_{k}$ of the background power level:
  (4) $$Z_{k}=\sum_{i=1}^{k}x\left(i\right)$$
6. Next, the corresponding scaling factor $T_{k}$ is selected based on the *k* and the desired probability of false alarm (PFA). The implementation method of $T_{k}$ is described in the next section.
7. Finally, a target present decision is made if the value of the test cell exceeds the adaptive threshold according to the hypothesis test:
  (5) $$H_{1}:x_{0}\geq T_{k}Z_{k};H_{0}:x_{0}<T_{k}Z_{k}$$
  Here, $H_{1}$ denotes the presence of a target in the test cell, while $H_{0}$ corresponds to the absence of a target in the test cell.

From the above algorithm flow, we can see that the FOD-CFAR method discussed in this paper has the following characteristics that distinguish it from other traditional CFAR algorithms.

1. In the automatic censoring process, the proposed CFAR detector does not require any prior knowledge about the background environment. It makes FOD-CFAR more suitable to a changing environment.
2. The detector discussed here employs a fixed detection threshold to reject interference targets, and it is not a cell-by-cell censoring procedure, which allows acceptance or rejection of the ordered cells by performing a successive threshold design. It can bring some benefits to computational efficiency.

## 4. FOD-CFAR Parameter Selection

Compared to other CFAR detectors, the performance of the FOD-CFAR is dependent on the values used for the threshold $\beta_{\alpha }$ and scaling factor $T_{k}$. This section gives the selection method of these parameters.

### 4.1. Threshold $\beta_{\alpha }$

From the algorithm flow in Section 3, we can see that the use of the hypothesis tests in Step 4 requires the values of the corresponding threshold $\beta_{\alpha }$. We use *p* lowest cells of ordered samples ${\bar{X}}_{N}$ to calculate the threshold $\beta_{\alpha }$. In order to obtain better censoring efficiency of FOD-CFAR, it is imperative to select the quantity of *p* properly. The authors [38] have derived the expression of the probability *γ* that the *p* lowest cells are homogeneous in either multiple target situations or a uniform background.

(6) γ=1-c1∑j=0p-1(-1)jp-1j(c2+j)(c3+j)

where:

(7) c1=pN-mpm1+INR,c2=N-m-p+1,c3=m1+INR+c2

*m* is the number of interference targets and INR stands for interference target-to-noise/clutter ratio. Table 1 gives some examples of probability *γ* obtained for different *m* and INR. We observe that the probability *γ* is close to one as INR increases and *m* decreases.

After determining the proper quantity of (N,p), then $\beta_{\alpha }$ is calculated using:

(8) $$\beta_{\alpha }=\alpha \sqrt{\frac{1}{p-1}\sum_{i=1}^{p}{\left(x\left(i\right)-\bar{x}\right)}^{2}}$$

where $\bar{x}$ is the arithmetic mean of the *p* lowest cells of ${\bar{X}}_{N}$. Formally, $\beta_{\alpha }$ is *α* times the standard deviation of *p* lowest cells of ${\bar{X}}_{N}$.

The selection of *α* requires that the values of corresponding thresholds $\beta_{\alpha }$ be determined to ensure the explicit probability of the hypothesis test error in a homogeneous environment. The hypothesis test error is defined as the probability of error $P_{error}$, such that a homogeneous environment is classified as variable:

(9) $$P_{error}=P_{rob}\left[{\bar{Y}}_{N-1}\geq \beta_{\alpha }|Homogeneous\;Environment\right]$$

Table 2 shows some examples of *α* as a function of $P_{error}$ and (N,p) in a homogeneous background. The *α* were obtained through Monte Carlo simulation. The number of simulation trials is 100,000.

**Figure 4:**
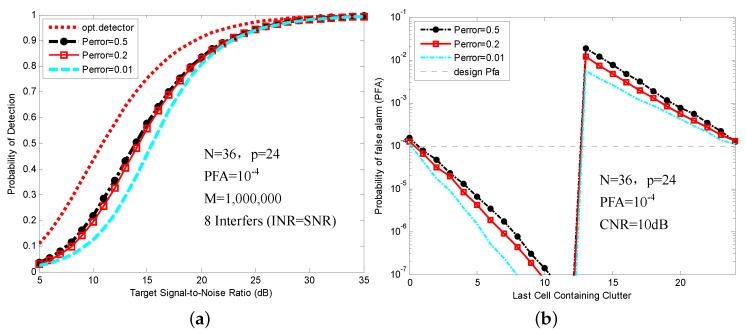
Probability of detection (PD) and probability of false alarm (PFA) values as a function of $P_{error}$: (**a**) PD comparison in a multiple target situation (eight interference targets); (**b**) PFA comparison in a clutter edge environment (CNR = 10 dB).

We observe that as the the $P_{error}$ steadily decreases, the *α* increases. Figure 4 shows the probability of detection (PD) in a multiple target situation and PFA regulation in a clutter edge environment by using different values of $P_{error}$. The explicit simulation parameters are shown in Section 5.

According to the simulation results, we find that selection of $P_{error}$ is a compromise between the detection probability of the multiple target situation and the false alarm regulation of the clutter edge environment. The result in Figure 4 shows that as $P_{error}$ decreases, the detection probability of the multiple target situation decreases and the false alarm regulation of the clutter edge environment increases. To balance the detection performance, $P_{error}$ is set to 0.2 in this paper. The simulation results in different clutter environments are shown in Section 5.

### 4.2. Scaling Factor

Once the upper end N-k cells are censored, the remaining *k* lowest samples are combined to form the statistic $Z_{k}$. Then, it is imperative to calculate the corresponding scaling factor $T_{k}$ to yield the FOD-CFAR threshold $T_{k}Z_{k}$. Using both the moment generating function and the contour integral, the authors [37] have derived the PFA in a homogeneous background when exactly N-k out of *N* cells are censored from the upper end.

(10) Pfa=Nj∏j=1k(Tk+N-j+1k-j+1),k>N2

Therefore, the background multiplier constant $T_{k}$ can be computed from Equation (10) for a specific $P_{fa}$.

## 5. Performance Results

We now study the performance of FOD-CFAR detector for a variety of homogeneous, multiple target and clutter edge environments. In different clutter environments, we first give the censoring probability of interference targets and then compare the detection performance of FOD-CFAR to those of the CA-CFAR, OS-CFAR, CMLD and VI-CFAR.

The PFA was set to $10^{-4}$. Each individual data point representing CFAR performance performs 1,000,000 simulation trials, which is marked as *M*. Detection analysis for Swerling II targets and single pulse processing is presented in this paper.

For the OS-CFAR, the value of $k_{OS}$ parameter was selected equal to 0.75N, which is the optimum censoring point [34]. For the CMLD, the number of largest cell censored from the reference window was set equal to four when (N,p) = (24,16) and was set equal to six when (N,p) = (36,24). Furthermore, we use VI threshold $K_{VI}=4.76$ and mean ratio threshold $K_{MR}=1.806$ for the VI-CFAR [21]. Specifically, the value *α* of FOD-CFAR was set equal to six when (N,p) = (24,16) and was set equal to 5.9 when (N,p) = (36,24), both of them yielding a $P_{error}$ of approximately 0.2.

### 5.1. Scenario 1: Homogeneous Environment

**Figure 5:**
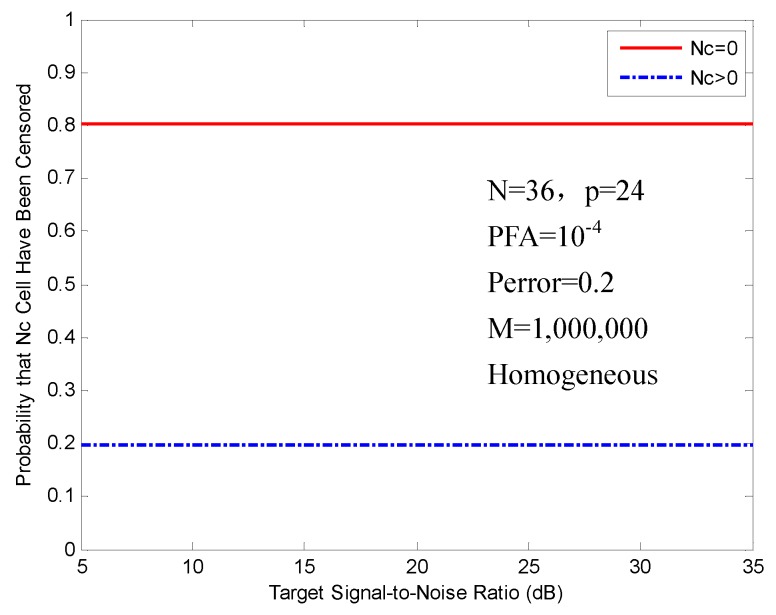
Probability of censoring in a homogeneous environment.

Figure 5 shows the probability of censoring for FOD-CFAR in a homogeneous environment. The (N,p) is set to (36,24). The probability that zero cells have been censored ($N_{c}=0$) equals 0.8 and corresponds to the case of homogeneous cell. The probability that greater than zero cells have been censored ($N_{c}>0$) is 0.2, which also equals $P_{error}$. Both of the censoring probabilities are constant in a homogeneous environment independent of the SNR of the target in the test cell.

**Figure 6:**
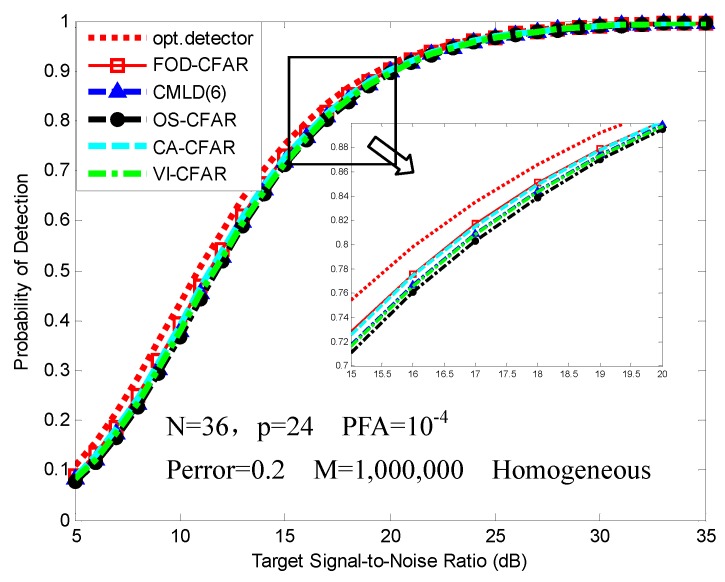
PD comparison of FOD-CFAR, censored mean level detector (CMLD)(6), order statistic (OS)-CFAR, cell-averaging (CA)-CFAR, variability index (VI)-CFAR in a homogeneous environment.

Figure 6 shows the PD in a homogeneous environment for different CFAR processors along with the result for an optimal detector (exactly known noise power). We observe that all of the CFAR detectors exhibit some CFAR loss relative to the optimal detector. The FOD-CFAR and CA-CFAR exhibit the same CFAR loss relative to the optimal detector. The CMLD and VI-CFAR are slightly worse than FOD-CFAR and CA-CFAR. The OS-CFAR exhibits the worst performance.

### 5.2. Scenario 2: Multiple Target Situations

**Figure 7:**
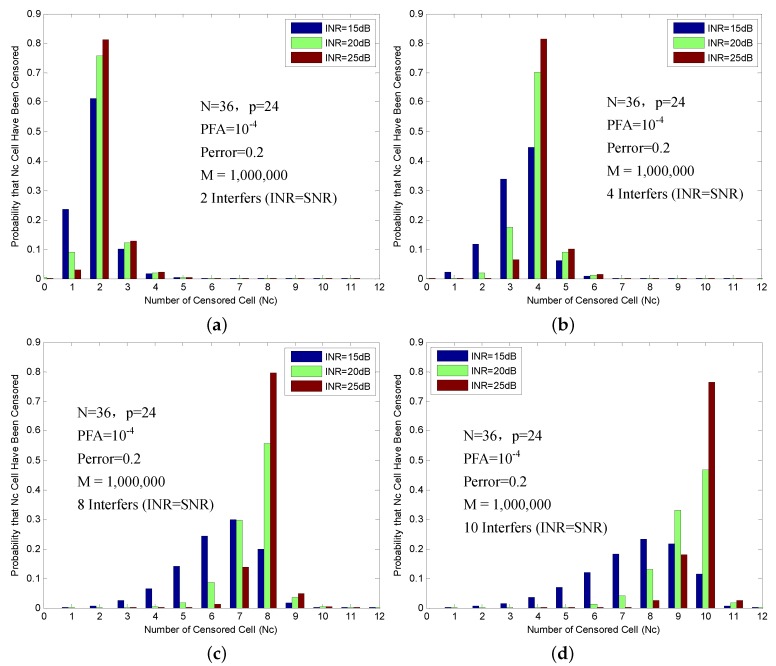
Probability of censoring in multiple target situations: (**a**) Two interference targets; (**b**) Four interference targets; (**c**) Eight interference targets; and (**d**) Ten interference targets.

Figure 7 shows the probabilities of censoring, which are obtained with 2, 4, 8 and 10 interfering targets of different INR. The interference targets are assumed to have the same radar cross-section (RCS) as the primary target, namely INR=SNR. Meanwhile, the interference targets are not confined to a single half of the reference window. The (N,p) is set to (36,24).

The $P_{censor}$ is defined as the probability of the explicit number of cells censored from the reference window. The $P_{censor}$ of the exact number of interference targets having been censored is summarized in Table 3. As expected, it is easy to find that $P_{censor}$ gets bigger as INR increases. It can also be observed that $P_{censor}$ grows smaller as the number of interferences increases.

**Figure 8:**
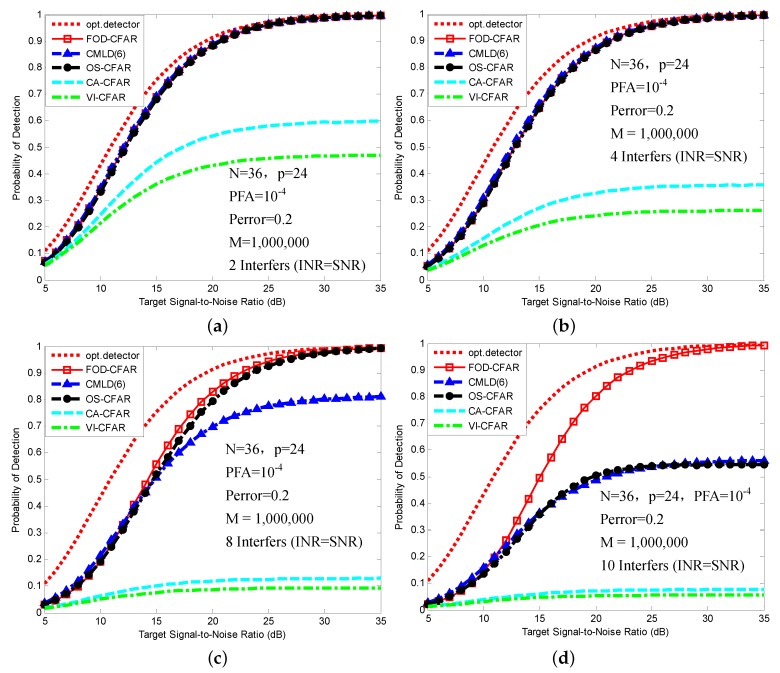
PD comparison of FOD-CFAR, CMLD(6), OS-CFAR, CA-CFAR and VI-CFAR in multiple target situations: (**a**) Two interference targets; (**b**) Four interference targets; (**c**) Eight interference targets; and (**d**) Ten interference targets.

In Figure 8, we present a comparison of the proposed FOD-CFAR with other CFAR processor in multiple target situations. We note that FOD-CFAR is robust in the sense that no substantial detection degradation occurs even for m=10. The excessive degradation in performance occurs when m≥2 for the CA-CFAR. Because interfering targets are not confined to a single half of the reference window, the excessive degradation in performance occurs when m≥2 for the VI-CFAR. Because of a mismatch between the preset number of censored cells and the actual number of interference targets, the excessive degradation in performance occurs when m≥8 for the CMLD(6). The excessive degradation in performance occurs when m=10 ($m>N-k_{os}=9$) for the OS-CFAR.

### 5.3. Scenario 3: Clutter Edge Environment

In the clutter edge environment, we assume that $N_{clutter}$ is the number of high clutter cells and $N-N_{clutter}$ is the number of low noise/clutter cells in the reference window. The test cell is assumed to be in the low noise/clutter power region if $N_{clutter}\leq \frac{N}{2}$, while it is in the high clutter region if $N_{clutter}>\frac{N}{2}$. Clutter edges progress from left to right of the reference window.

We first analyze the probabilities of censoring for the FOD-CFAR in the clutter edge environment. The clutter-to-noise ratio (CNR) is set to 20 dB, and (N,p) is set to (24,16).

**Figure 9:**
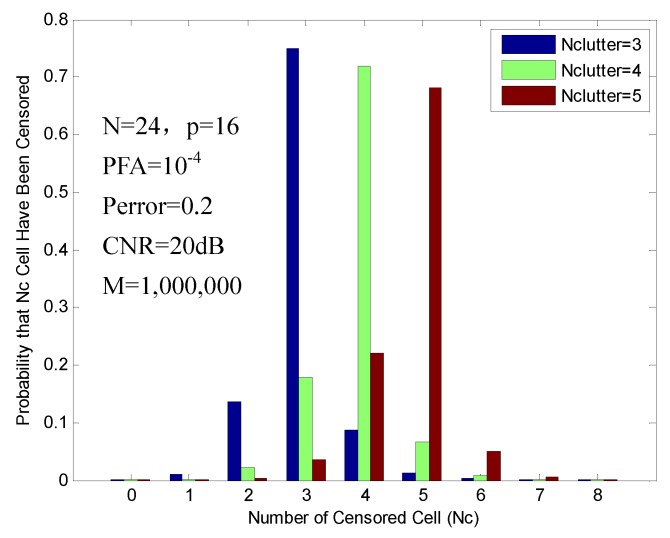
Probability of censoring in the clutter edge environment (3, 4 and 5 clutter cells).

As the clutter first enters the reference window, for $N_{clutter}\leq \frac{N}{2}=12$, $N_{clutter}\leq N-p=8$, the performance of censoring is similar to that of multiple target situations. Figure 9 shows the probabilities of censoring, which are obtained with 3, 4 and 5 clutter cells in the reference window. In this situation, the algorithm rejects the exact number of interferences ($N_{c}$ = 3, 4, 5) for 74.89%, 71.96% and 68.26% of the time, respectively. FOD-CFAR exhibits good censoring performance in this situation.

**Figure 10:**
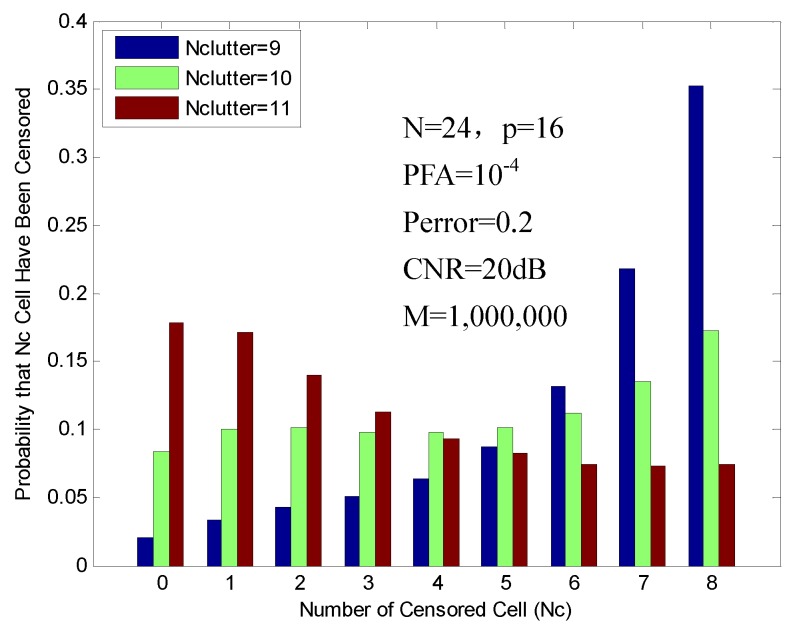
Probability of censoring in the clutter edge environment (9, 10 and 11 clutter cells).

As the high clutter continues to move into the reference window, for $N_{clutter}\leq \frac{N}{2}=12$, $N_{clutter}>N-p=8$, due to the fact that the censoring procedure of FOD-CFAR does not reject more than N-p high clutter cells, the remaining $N_{clutter}-\left(N-p\right)$ high clutter cells may cause substantial censoring degradation. Figure 10 shows the probabilities of censoring, which are obtained with 9, 10 and 11 high clutter cells in the reference window. In this figure, the algorithm rejects the N-p high clutter cells ($N_{c}$ = 8) for 35.25%, 17.24% and 7.46% of the time, respectively. In this case, the poor censoring performance of high clutter will cause relative degradation in the detection performance.

**Figure 11:**
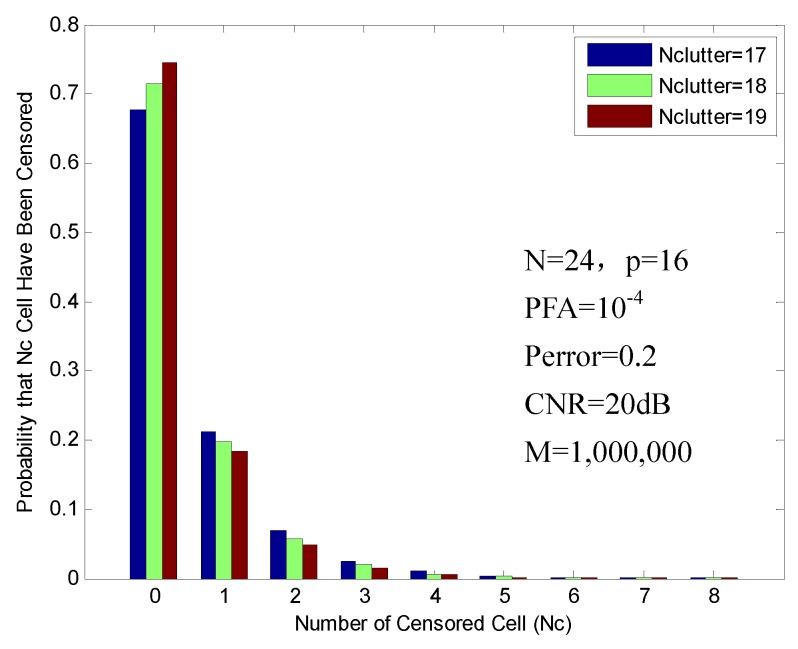
Probability of censoring in the clutter edge environment (17, 18 and 19 clutter cells).

Finally, for $N_{clutter}>\frac{N}{2}=12$, due to the fact that the test cell is in the high clutter, the number of high clutter cells to be censored must be as small as possible to prevent an excessive rise in PFA. At best, none of the high clutter cells will be censored. Figure 11 shows the probabilities of censoring, which are obtained with 17, 18 and 19 clutter cells in reference window. In this figure, the probabilities that zero high clutter cells have been censored ($N_{c}$ = 0) are 67.63%, 71.53% and 74.57% of the time, respectively. In this case, the high clutter cells are well reserved. However, the remaining low clutters will cause a relative increase in the PFA.

**Figure 12:**
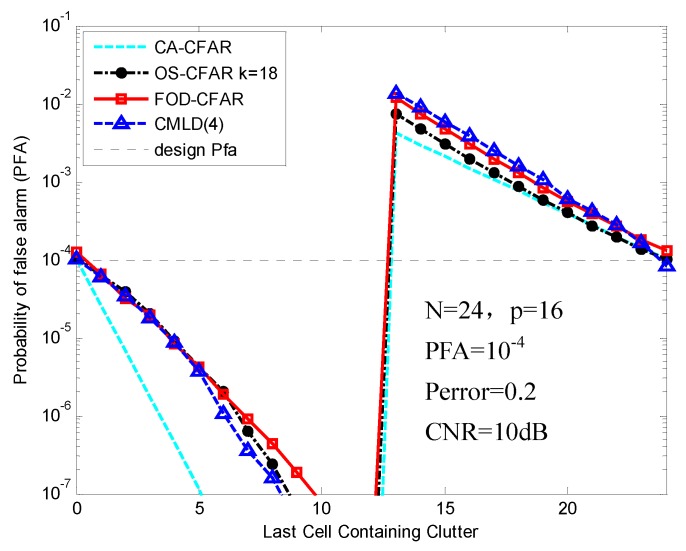
PFA comparison of FOD-CFAR, OS-CFAR, CA-CFAR and CMLD(4) in the clutter edge environment.

Figure 12 shows the PFA of different CFAR processors in the clutter edge environment where the CNR is 10 dB. Note that the PFA regulation of the FOD-CFAR is slightly worse than the traditional OS-CFAR and CA-CFAR and is slightly better than CMLD(4).

### 5.4. Time Complexity Analysis

Because the uniqueness of FOD-CFAR with respect to other CFAR detectors lies in how the background power estimation is performed, we only give the time complexity of background power estimation. As an order statistics-based CFAR detector, the reference cells are first ranked to form the ordered samples in FOD-CFAR. We use the quick sort algorithm in this paper, whose time complexity is:

(11) $$C_{rank}=O\left(N\log_{2}N\right)$$

After the time complexity of the followed automatic censoring process is added to Equation (11), the total time complexity of FOD-CFAR is:

(12) $$C_{FOD-CFAR}=C_{rank}+3N+4p-2n+1=O\left(N\log_{2}N\right)$$

where *n* is the number of censored cells, 0≤n≤N-p. From Equation (12), we can see that the time complexity of FOD-CFAR mainly comes from $C_{rank}$, and the time complexity of the followed automatic censoring process is only O(N). On the basis of sorting the reference cells, FOD-CFAR only suffers a small loss of time complexity to obtain a better detection performance in nonhomogeneous clutter environments.

### 5.5. Experimental Results

**Figure 13:**
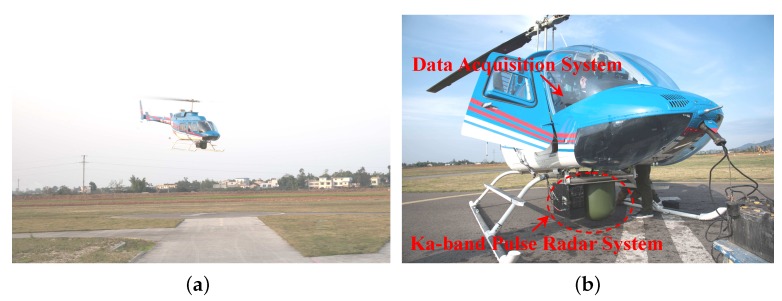
Experimental test platform: (**a**) Overview of the helicopter; (**b**) Onboard radar system.

This section is devoted to the performance assessment of the new algorithms in the presence of real data. We performed a flight testing in Luodai Town, Chengdu, to collect the radar measured data used for developing and testing our detection algorithm. The flight test was held in December 2015. Figure 13a shows a sketch of our test platform. The helicopter used to test is Bell-407. Figure 13b shows an overview of onboard radar system. The system is a Ka-band pulse radar and mainly consists of an antenna, turntable, transmitter, receiver and signal acquisition system.

**Figure 14:**
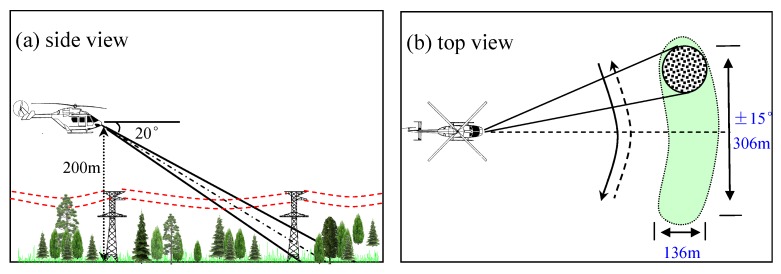
Experimental flight pattern. (**a**) Side view; (**b**) Top view.

Figure 14 shows the flight pattern of this experiment. The flight altitude is set to 200 m; the pitch angle is set to $20^{\circ }$, and the scan coverage is set to ±$15^{\circ }$. The radar scans the coverage in 0.5 s in a continuous azimuth scan mode. According to the designed flight pattern, the theoretical azimuth coverage is 306 m and is 136 m in the range direction. The flight pattern is designed to better simulate the target environment of the low-flying helicopter, while guaranteeing the flight safety by a pilot without special low-flying training.

The signal parameters are listed in Table 4.

**Figure 15:**
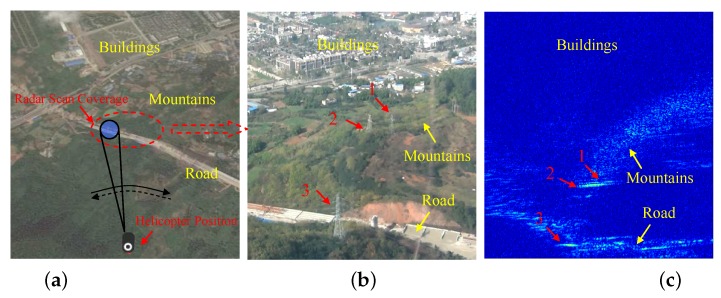
Data collection scene: (**a**) Latitude and longitude information of the low-flying helicopter corresponding to the chosen target scene marked on Google Maps; (**b**) Optical image corresponding to the radar scan coverage; (**c**) Corresponding radar data matrix.

Figure 15 shows a single frame of the target scene and its corresponding radar echo matrix in this experiment. First, we use latitude and longitude information recorded in the inertial navigation system (INS) and time synchronization information recorded in the receiver to find the corresponding optical image and radar echo matrix. The latitude and longitude information of the low-flying helicopter corresponding to the chosen target scene are marked on the Google Maps, which is shown in Figure 15a. The corresponding optical image and echo matrix are shown in Figure 15b,c, respectively. From the Figure 15b, we can see that there are many artificial targets, such as power towers on the flight path of the low-flying helicopter. These obstacles will pose a significant challenge to the helicopter if the flight altitude is further decreased. In Figure 15c, the radar echo matrix consists of 1400 range cells and 2240 azimuth samples and is displayed in a B-scope plot. The samples in the data matrix are the output of a squared-law detector. Data in the same row of the echo matrix represent samples in the azimuth direction, whereas data in the same column represent samples in the range direction.

From Figure 15b,c, we can see that there is a corresponding relationship between the road-like artificial targets and the mountainous areas with rapid elevation variation. Furthermore, because the power tower mainly consists of angle iron, the radar cross-section (RCS) of the power tower is often bigger than the background, which mainly consists of trees and concrete. For these reasons, we recognize that the three parts of the strong scatterers in the radar data matrix are due to the power tower. Meanwhile, because the radar scan coverage in the range direction (also shown in Figure 14b) is limited by the vertical beam width, the buildings behind Target 1 and Target 2 in Figure 15b do not exist in the radar echo matrix in this frame.

**Figure 16:**
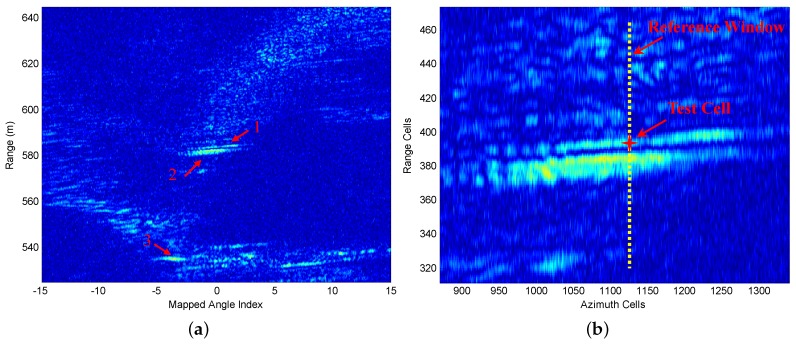
Two styles of zoom-in views of the radar data matrix: (**a**) B-scope image display of the data matrix; (**b**) Zoom-in view of Target 1 and Target 2.

Figure 16 offers two styles of zoom-in views of the radar data matrix. From Figure 16, we can see that Target 1 and Target 2 are close to each other in the range direction. Therefore, the two targets can simulate the multiple target situation of a nonhomogeneous clutter environment. Meanwhile, due to the fluctuation of their background terrain, there is also a clutter edges situation in the radar echo matrix.

**Figure 17:**
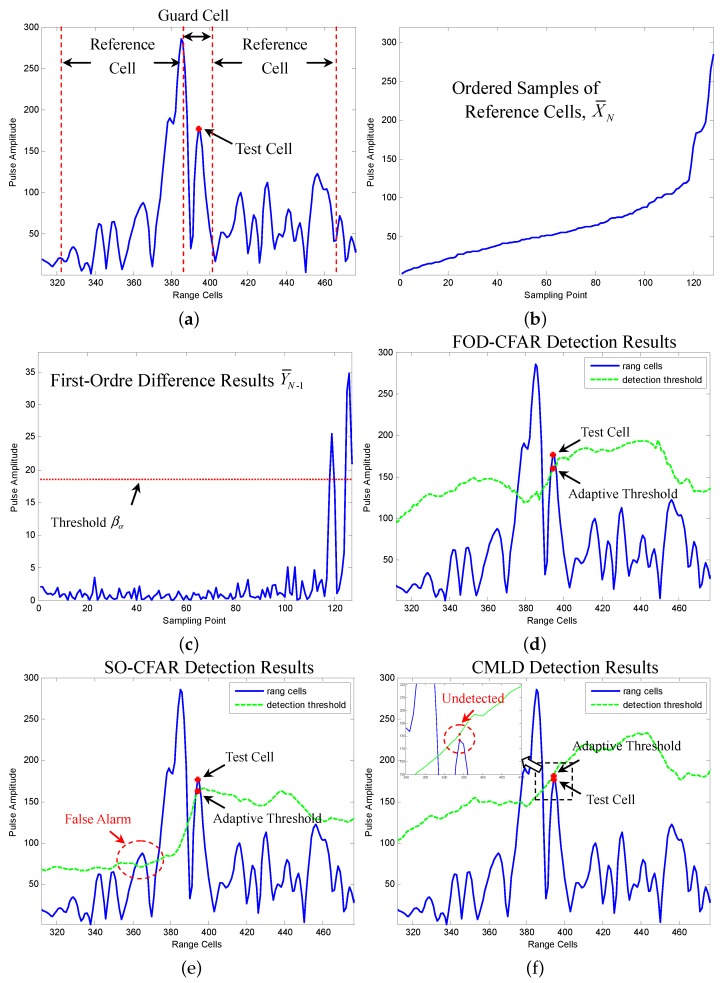
Performance comparison of FOD-CFAR, SO-CFAR and CMLD on a single resolution cell: (**a**) Reference window of the chosen resolution cell; (**b**) Ordered samples ${\bar{X}}_{N}$ of the reference window; (**c**) First-order difference results ${\bar{Y}}_{N-1}$ and the threshold $\beta_{\alpha }$; (**d**) FOD-CFAR detection results; (**e**) SO-CFAR detection results; (**f**) CMLD detection results.

The FOD-CFAR detector is performed at each resolution cell using a sliding window along each column of the data matrix. The (N,p) is set to (128,85). The guard cell is set to 16. The PFA was set to $10^{-1}$, and $P_{error}$ is set to 0.97. Figure 17 illustrates the target detection performance of FOD-CFAR on a single resolution cell. The chosen resolution cell and its reference window are also marked on Figure 16b. Figure 17a shows the reference window of the chosen resolution cell. Corresponding to the algorithm flow in Section 3, Figure 17b gives the ordered samples ${\bar{X}}_{N}$ of the reference cells. Figure 17c illustrates the first-order difference results ${\bar{Y}}_{N-1}$ and its threshold $\beta_{\alpha }$. In Figure 17c, we find that ${\bar{Y}}_{N-1}\left(118\right)$ is greater than $\beta_{\alpha }$. We decide that the ordered subset [x(1)≤x(2)≤⋯≤x(118)] corresponds to homogeneous returns. After censoring the 128-118 highest ranked cells, the remaining 118 samples are combined to form an estimate $Z_{k}$ of the background power level. Afterwards, the corresponding scaling factor $T_{k}$ is selected based on the 118 and the desired PFA. Figure 17d gives the FOD-CFAR detection results on the chosen resolution cell. We can see that the test cell exceeds the adaptive threshold $T_{k}Z_{k}$. Meanwhile, the detection thresholds of other resolution cells are also plotted in Figure 17d, which is denoted with the dotted line.

In order to better illustrate the detection performance, we present a comparison of the proposed FOD-CFAR with SO-CFAR and CMLD on the chosen resolution cell. For the CMLD, the number of largest cells censored from the reference window was set equal to 30. Figure 17e gives the SO-CFAR detection results on the chosen resolution cell. We can see that the test cell exceeds the adaptive threshold. However, the plot shows that around Range Cell 365, there is a great concentration of false alarms. Figure 17f shows the CMLD detection results on the chosen resolution cell. We can see that the test cell is below the adaptive threshold and is undetected. The above results show that FOD-CFAR exhibits robust detection performance on the chosen resolution cell, which is better than classic SO-CFAR and CMLD.

**Figure 18:**
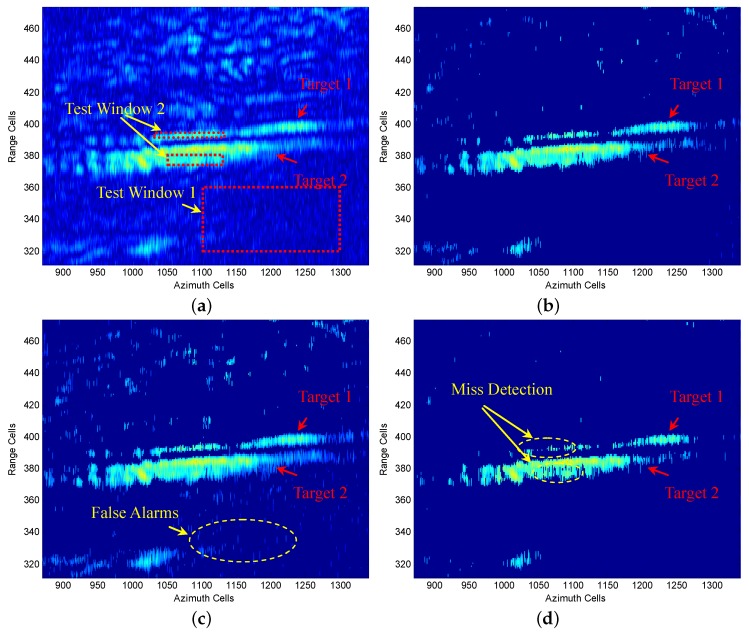
Performance comparison of FOD-CFAR, SO-CFAR and CMLD on the chosen scene: (**a**) Zoom-in view of Target 1 and Target 2; (**b**) FOD-CFAR detection result; (**c**) SO-CFAR detection result; (**b**) CMLD detection result.

Furthermore, we present a comparison of the proposed FOD-CFAR with SO-CFAR and CMLD on the chosen target scene shown in Figure 16b. The original target scene is also given in Figure 18a. The data matrix in Figure 18a consists of 163 range cells and 471 azimuth samples. From Figure 18b, it can be seen that both Target 1 and Target 2 are well detected based on FOD-CFAR detector in the real nonhomogeneous clutter environment. Figure 18c shows the SO-CFAR detection result on the chosen target scene. We can see that both Target 1 and Target 2 are well detected. However, the same as the detection result shown in Figure 17e, the main drawback of SO-CFAR is the excessive number of false alarms. Figure 18d gives the CMLD detection results on the chosen target scene. The same as the detection result shown in Figure 17f, we can see that the main drawback of the CMLD is the excessive number of misdetections. The results show that FOD-CFAR exhibits robust detection performance on the chosen target scene, which is better than classic SO-CFAR and CMLD.

Finally, we are devoted to a quantitative performance analysis of the FOD-CFAR, SO-CFAR and CMLD in the presence of real data. The experiment is conduct on the chosen target scene shown in Figure 18a. In order to complete the performance analysis, three assessment criteria are defined, namely the number of false alarms (NFA), the number of misdetections (NMD) and the response time (RT).

First of all, the NFA is determined by counting the number of targets falling into Test Window 1. Test Window 1 is shown in Figure 18a and consists of 40 range samples and 200 azimuth samples. The total number of cells available for estimating the NFA is 8000.

Second, the NMD is calculated by counting the number of undetected targets falling into Test Window 2. Test Window 2 is shown in Figure 18a. Test Window 2 consists of two subwindows. The fist one consists of three range samples and 100 azimuth samples, and the second one consists of eight range samples and 65 azimuth samples. The total number of cells available for estimating the NMD is 820.

Third, the RT is calculated by the elapsed time of the FOD-CFAR, SO-CFAR and CMLD detectors. The FOD-CFAR, SO-CFAR and CMLD detection results are shown in Figure 18b–d, respectively. All of the RT experiments are running on the MATLAB software and are accomplished with a hardware environment of Inter Core 3.2 GHz CPU and 8 GB memory.

The NFA, NMD and RT are summarized in Table 5. The same as the detection result shown in Figure 17 and Figure 18, we can see that the main drawback of SO-CFAR is the excessive NFA, and the main drawback of the CMLD is the excessive NMD. The result leads to the conclusion quantitatively that FOD-CFAR exhibits better detection performance than SO-CFAR and CMLD. Meanwhile, we must point out that the RT of FOD-CFAR is bigger than SO-CFAR and CMLD, which is due to the added automatic censoring process proposed in this paper.

However, in the experimental results, we also find that all of the CFAR detectors are not able to maintain rigorously the theoretical false alarm, and the selection of $P_{error}$ of FOD-CFAR does not match with the values used in the simulation. The reason for that is that the independent and identical Gaussian distributed assumption is not always valid in the real data [26,41]. In order to obtain better detection performance of real data, possible research tracks might extend the proposed automatic censoring technique to the non-Gaussian clutter distributed assumption.

## 6. Conclusions

In this paper, a new automatic censoring CFAR detector named FOD-CFAR has been proposed and tested in order to improve the radar signal detection performance of a low-flying helicopter in nonhomogeneous clutter environments. The proposed FOD-CFAR detector does not require any prior information about the background environment and uses the hypothesis test of the FOD result of the ordered data to reject the unwanted samples in the reference window. The simulation results show that the FOD-CFAR offers low-loss CFAR performance relative to the optimal detector in a homogeneous environment. Meanwhile, FOD-CFAR exhibits robust detection performance in multiple target situations even when 10 interference targets exist in a 36 length reference window, which is better than CA-CFAR, OS-CFAR, VI-CFAR and CMLD. In clutter edge environments, the PFA regulation of the FOD-CFAR is slightly worse than CA-CFAR and OS-CFAR. Furthermore, on the basis of the sorting of the reference cells, the time complexity of the proposed automatic censoring process is only O(N). Finally, The measured result leads to the conclusion quantitatively that FOD-CFAR exhibits better detection performance than SO-CFAR and CMLD.

The FOD-CFAR can help radar get a better detection performance to obstacles in nonhomogeneous clutter environments, thus further improving the safety and environment adaptability of a low-flying helicopter. Possible future research tracks might concern the performance improvement of PFA regulation in a clutter edge environment and extend the proposed automatic censoring technique to a non-Gaussian clutter distributed assumption [42,43,44,45] and Weibull [46] or Swerling-Chi [47] fluctuating targets.

## Figures and Tables

**Table 1:** Probability *γ* that *p* lowest cells are homogeneous in the interference environment.

(N,p)	*m*	INR				
		15dB	20dB	25dB	30dB	40dB
(24,16)	2	0.9269	0.9757	0.9922	0.9975	0.9998
	4	0.8316	0.9419	0.9811	0.9940	0.9994
	8	0.4554	0.7687	0.9187	0.9734	0.9973
(36,24)	4	0.8490	0.9483	0.9832	0.9947	0.9995
	8	0.6398	0.8646	0.9546	0.9854	0.9985
	12	0.2735	0.6454	0.8679	0.9559	0.9955

**Table 2:** *α* values as a function of $P_{error}$, *N* and *p*.

(N,p)	$P_{error}$						
	0.5	0.4	0.3	0.2	0.1	0.05	0.01
(24,16)	3.2	3.9	4.8	6.0	8.2	10.7	16.9
(36,24)	3.2	3.8	4.7	5.9	8.1	10.4	16.3

**Table 3:** Probability $P_{censor}$ that the exact number of interference targets has been censored.

Interference Cell	Censored Cell ($N_{c}$)	INR		
		15 dB	20 dB	25 dB
2	2	61.13%	75.70%	81.22%
4	4	44.73%	70.02%	81.49%
8	8	19.91%	55.78%	79.53%
10	10	11.40%	46.65%	76.54%

**Table 4:** Experimental parameters.

General Parameters	Value
Frequency	35 GHz
Flight Speed	30 m/s
Flight Altitude	200 m
Pitch Angle	20 ${}^{\circ }$
Scan Coverage	±15${}^{\circ }$
Horizontal Beam Width	4${}^{\circ }$
Vertical Beam Width	4.5${}^{\circ }$
Antenna Scan Rate	60${}^{\circ }$/s
Signal Bandwidth	200 MHz
Pulse width	1 μs
PRF	4000 Hz

**Table 5:** Performance analysis of the FOD-CFAR, SO-CFAR and CMLD on the real data.

CFAR Detector	Number of False Alarms (NFA)	Number of Misdetections (NMD)	Response Time (RT)
SO-CFAR	197	154	0.81 s
CMLD	0	427	0.98 s
FOD-CFAR	0	246	2.44 s
Total Number	8000	820	
